# Are Readers’ Eyes Worth Saving?

**Published:** 1899-04

**Authors:** D. Albert Hiller

**Affiliations:** 220 Montgomery Ave., San Francisco, Cal.


### ARE READERS' EYES WORTH SAVING?

EDITOR HOMŒOPATHIC PHYSICIAN: I wish to congratulate you in regard to the fine paper and large, clear, and readable print, furnished in your journal.

You have refrained from falling into the late fad of printing on that abominable, corduroy-ribbed paper which sub-divides the printed letters and makes ordinary medical journals so hard to read.

This kind of ribbed paper (and consequent poor print) is very injurious to the eyes and often compels the reader to discontinue not only his reading but his subscription. Years ago Professor Gustav Jager drew attention to the necessity for sanitary paper and print in all public journals, and it is surprising that some publishers should pay little or no attention to this matter, and should so entirely ignore the welfare of their subscribers, so far as eye-sight is concerned.

Many subscriptions have undoubtedly been discontinued because of such really criminal neglect of ordinary precautions, and some *otherwise good* journals have gone to the wall on that account.

This is a matter of much moment, for many of the prints of the day in all lines of literature are similarly destructive of the readers' eyes, and it is high time that some one should bring attention in a notable manner, to this very dangerous defect. The Homœopathic Physician is in the front rank of homœopathic literature, and I sincerely hope that you will continue to be aided by the progressive physicians of our school, who have so little time to spare in reading and keeping up with the times in their profession, and who cannot afford to sacrifice their eyes, in order that they may obtain information, contained in other journals of the day. I can read and properly digest the valuable articles published in The Homœopathic Physician in half the time taken up with any other publication of the kind, of equal size, and can at the same time turn labor into pleasure in reading its plainly printed pages and enjoying its valuable contents. Hence, I think that the general appearance of your publication, as well as other considerations, make it one to be commended as a pattern for other medical journals to follow.

Wishing you all success, I am,

Fraternally yours,

D. Albert Hiller.

220 Montgomery Ave., San Francisco, Cal., March 28th, 1899.

